# Non-phosphorylated Tyr-1248 form of human epidermal growth factor receptor 2 (HER2) predicts resistance to trastuzumab therapy and poor disease-free survival of HER2-positive breast cancer patients

**DOI:** 10.3325/cmj.2022.63.126

**Published:** 2022-04

**Authors:** Snježana Ramić, Frane Paić, Velda Smajlbegović, Melita Perić Balja, Lea Hiršl, Ingrid Marton, Fabijan Knežević

**Affiliations:** 1 Department of Oncological Pathology, University Hospital for Tumors, Sestre Milosrdnice University Hospital Center, Zagreb, Croatia; 2 Laboratory for Epigenetic and Molecular Medicine, Department of Medical Biology, University of Zagreb School of Medicine, Zagreb, Croatia; 3 Oncology Clinic, Clinical Center of Sarajevo University, Sarajevo, Bosnia and Herzegovina; 4 Center for Proteomics, Faculty of Medicine, University of Rijeka, Rijeka, Croatia; 5 Department of Pathology, Sveti Duh University Hospital, Zagreb, Croatia

## Abstract

**Aim:**

To determine the predictive value of phosphorylated human epidermal growth factor receptor 2 (pHER2^Y1248^) status in breast cancer (BC) patients undergoing trastuzumab-based adjuvant therapy.

**Methods:**

Immunohistochemical status of pHER2^Y1248^, EGFR/HER1, HER3, and HER4 was determined in 124 consecutive HER2-positive BC patients (median age [range] = 57 years [49.0-64.0]) treated at the University Hospital for Tumors, Zagreb, between 2008 and 2011. The median follow-up was 84 months (60.0-84.0). Prognostic factors of disease free survival (DFS) rate were evaluated with Kaplan-Meier/log-rank test and Cox regression analysis.

**Results:**

pHER2^Y1248^, HER1, HER3, and HER4 were expressed in 66.1%, 9.7%, 70.2%, and 71.0% of patients, respectively. Disease progression (DP) was observed in 17.1% of pHER2^Y1248^-positive and 47.6% of pHER2^Y1248^-negative BCs (*P* = 0.001). Kaplan-Meier analysis showed a worse five-year DFS in pHER2^Y1248^-negative patients who were older than 60 years (*P* < 0.001) and had positive lymph node status (*P* < 0.001); tumor size >2.0 cm (*P* < 0.001); higher histological grade (*P* < 0.001); HER2E intrinsic subtype (*P* < 0.001), negative hormone receptors (*P* < 0.001); negative HER1 status (*P* < 0.001), positive HER3 (*P* = 0.002); and/or positive HER4 (*P* = 0.002) status. The only negative prognostic factor for five-year DFS in multivariate Cox regression analysis was pHER2^Y1248^-negative (hazard ratio [HR] 3.6, 95% confidence interval [CI] 1.8-7.2, *P* < 0.001) and lymph node-positive status (HR 3.6, 95% CI 1.3-9.8, *P* = 0.014).

**Conclusion:**

pHER2^Y1248^ predicts sensitivity to trastuzumab and a better five-year DFS regardless of any other prognostic parameter. In HER2-positive BC patients. Non-phosphorylated HER2^Y1248^ is a strong predictor of trastuzumab resistance and a poor DFS.

Until the development of trastuzumab, a highly-specific monoclonal antibody targeted against human epidermal growth factor receptor 2 (HER2), breast cancer (BC) with positive HER2 was an aggressive and rapidly proliferating malignancy with a poor prognosis. HER2 is overexpressed in about 20% of BC patients, and trastuzumab reduces the risk of disease recurrence almost by half (1-4). However, a subset of HER2-positive BC patients fails to benefit from such therapy (1-6). Resistance to trastuzumab-based therapy was recorded in almost 30% of patients, and different resistance mechanisms have been described (1-7). Besides, HER2-positive BC is very heterogeneous and includes tumors with positive (luminal type) and those with negative (HER2-enriched type) estrogen- (ER) and progesterone- (PgR) hormone receptor status (5). Thus, the approach to BC treatment is not uniform, and the equal response to therapy or the same mechanisms of resistance cannot be expected (4-7). HER2 is a member of the epidermal growth factor receptor (ErbB) family of four human receptor tyrosine kinases (ErbB1/EGFR/HER1, ErbB2/HER2, ErbB3/HER3, and ErbB4/HER4). Ligand binding on the extracellular receptor domain leads to its homo- or hetero-dimerization, resulting in its activation/phosphorylation on the receptors' cytoplasmic domain (4,6,8,9). Although without a known ligand, HER2 is the preferred and the most potent dimerization partner due to its high catalytic activity. Active HER2 has several phosphorylation sites. In cases of overexpression (due to gene amplification as in HER2-positive BC), tyrosine-1248 (pHER2^Y1248^) is the most potent site because it is constitutively activated as a consequence of HER2 homodimerization. Other phosphorylation sites are usually activated by heterodimerization (6,8,9). So far, HER2 status is the only validated biomarker for anti-HER2 therapy in BC patients (1-3). However, detection of gene amplification or protein overexpression may not truly reflect the activated status of HER2. We assumed that the phosphorylation status of HER2 was a true indicator of its activity and that its heterodimerization with other ErbB family members might contribute to trastuzumab resistance. Therefore, the study aimed to evaluate the predictive value of pHER2^Y1248^ coexpressed with other ErbB family members and hormone receptors in HER2-overexpressing BC patients postoperatively treated with trastuzumab-based therapy.

## Patients and methods

### Patients

This retrospective study was performed on treatment-naive, archived formalin-fixed paraffin-embedded (FFPE) tumor tissues surgically removed from 124 consecutive patients diagnosed with HER2-positive primary ductal invasive breast cancer (BC). The patients were treated at the University Hospital for Tumors, Zagreb, between 2008 and 2011. All patients received adjuvant trastuzumab-based therapy for at least one year. Demographic and clinicopathological data were retrieved from medical records. Disease-free survival (DFS) rate was defined as the time in months from the date of surgery to the date of disease progression (DP). Data on disease progression, revealed by radiological methods (ultrasound, magnetic resonance, or positron emission tomography–computed tomography) as local recurrence or distant metastases, were obtained from the clinical database. Patients with other complications during trastuzumab therapy were not included. The follow-up period was over 84 months with the last check-up performed in May 2018. All participants signed the informed consent. The study was approved by the Ethics Committee of the University Hospital for Tumors (EP-15506/11-6).

### Immunohistochemical staining

pHER2^Y1248^ and ErbB family members were immunohistochemicaly analyzed on FFPE samples prepared as tissue microarray blocks (Tissue-Tek®Quick Ray System; Sakura, Japan). All BC samples are routinely processed immediately after surgery to avoid the potential loss of epitopes due to a delayed time to fixation. FFPE blocks were processed as previously described (10). Shortly, 3-μm thick serial microarray tissue sections were heated in a water bath for 20 min at 97 °C in an antigen retrieval solution, pH 9.0 (S2367; Dako, Glostrup, Denmark). The sections were incubated (overnight, 4 °C) with primary antibodies (pHER2^Y1248^: clone PN2A [Dako, Glostrup, Denmark] dilution 1:25; EGFR/HER1: clone E30 [Dako], dilution 1:50; HER3: clone DAK-H3-IC [Dako], dilution 1:50; HER4: clone sc-283 [Santa Cruz Biotechnology, Inc., Texas, USA], dilution 1:40). Subsequently, the sections were incubated (45 minutes, room temperature [RT]) with secondary antibody conjugated with horseradish peroxidase (EnVision Flex/HRP High pH; Dako) followed by incubation (10 minutes, RT) in DAB chromogen (3,3′-diaminobenzidine; Dako) and counterstained with hematoxylin.

The expression of pHER2^Y1248^ was assessed according to the semiquantitative HercepTest scoring method (11,12). BCs expressing moderate to strong membranous staining in more than 10% of cells were considered pHER2^Y1248^-positive (10). Tumors without staining, with weak membranous staining, or with only cytoplasmic staining were considered pHER2^Y1248^-negative.

The HercepTest method was also applied for immunohistochemical analysis of EGFR/HER1, HER3, and HER4 with BCs exhibiting 2+ or 3+ membranous/cytoplasmic staining considered as positive.

### Statistical analysis

The normality of distribution was assessed with the Shapiro-Wilk test. Data are presented as frequencies or median and interquartile range (IQR). Relationships between immunohistochemical data and clinicopathological parameters were assessed with the Spearman correlation (r), *t* test, and χ^2^, or Fisher exact test. The Kaplan-Meier/log-rank test was used to assess the difference in five-year DFS rate between the patient subgroups. Univariate and multivariate Cox proportional hazards model was used to determine the independent prognostic effect of individual variables on DFS rate, with the results presented as hazard ratio (HR) and 95% confidence interval (CI). All statistical tests were two-sided. The intergroup differences with α<0.05 were considered significant and corrected according to the Bonferroni procedure (the corrected level of significance is Pc =  0.05/N; N- number of independent tests). Statistical analysis was performed with SPSS, trial version (IBM Corp., Armonk, NY, USA).

## RESULTS

### Patients’ characteristics

The median age at the time of surgery was 57 years. The majority of patients were younger than 60 years (62.1%). Tumors >2.0 cm were present in 59.7% of patients. BC samples were mostly classified as histological grade III (56.45%) and were associated with positive axillary lymph nodes (60.5%) at the time of surgery (Table 1).

**Table 1:** Demographic and clinical characteristics of HER2-positive breast cancer (BC) patients before adjuvant trastuzumab-based therapy*

Characteristic	N (%)
Follow-up period; months, median (IQR)	84 (60.0-84.0)
Age of patients; years, median (IQR)	57 (49.0-64.0)
Age (years)	
<60	77 (62.1)
≥60	47 (37.9)
Tumor size; cm, median (IQR)	2.1 (1.6-3.0)
Tumor size stratification (cm)	
<2.0	50 (40.3)
≥2.0	74 (59.7)
Histological grade	
II	54 (43.5)
III	70 (56.4)
Estrogen receptor	
positive	67 (54.0)
negative	57 (46.0)
Progesterone receptor	
positive	50 (40.3)
negative	74 (59.7)
Intrinsic subtypes	
luminal B	70 (56.5)
HER2E	54 (43.5)
Lymph node status	
positive	75 (60.5)
negative	49 (39.5)
Disease progression	
present	34 (27.4)
absent	90 (72.6)

*Abbreviations: HER2 – human epidermal growth factor receptor 2; luminal B – estrogen receptor and/or progesterone receptor-positive and HER2 positive; HER2E – estrogen receptor and/or progesterone receptor-negative and HER2-positive intrinsic subtype; IQR – interquartile range.

The median follow-up was 84 months, and 34/124 (27.4%) patients had disease progression (DP). Among them, the median time to DP was 23.5 months (IQR 18.0-34.5). Positive expression of ER and PgR was detected in 54% and 40.3% of patients, respectively (Table 1). Thus, patients were classified in two intrinsic subtypes: luminal B (ER- and/or PgR-positive) and HER2E (ER- and PgR-negative), with 56.5% of patients belonging to the luminal B subtype (Table 1).

### Immunohistochemical expression of pHER2 and ErbB family members

The pHER2^Y1248^-positive staining was detected in 82 patients (2+ and 3+ staining status was 29.8% and 36.3%, respectively, Table 2). Although 42 (33.9%) patients were considered pHER2^Y1248^-negative, a total absence of staining was observed in only 9 (7.2%) patients.

**Table 2:** Association of pHER2 and other epidermal growth factors (ErbB) family members with clinicopathological prognostic features of breast cancer (BC) patients. The values are presented as frequencies and percentages*

	pHER2			ErbB1/HER1			ErbB3/HER3			ErbB4/HER4		
Variable	negative N = 42 (33.9)	positive N = 82 (66.1)	P^†^	negative N=112 (90.3)	positive N = 12 (9.7)	P^†^	negative N = 37 (29.8)	positive N = 87 (70.2)	P^†^	negative N = 36 (29.0)	positive N = 88 (71.0)	P^†^
Age of patients (years)												
<60	19 (45.2)	58 (70.7)	0.007	70 (62.5)	7 (58.3)	1.000	19 (51.4)	58 (66.7)	0.156	20 (55.6)	57 (64.8)	0.415
≥60	23 (54.8)	24 (29.3)		42 (37.5)	5 (41,7)		18 (48.6)	29 (33.3)		16 (44.4)	31 (35.2)	
Size of tumor (cm)												
<2.0	18 (42.9)	32 (39.0)	0.703	46 (41.1)	4 (33.3)	0.761	16 (43.2)	34 (39.1)	0.693	16 (44.4)	34 (38.6)	0.687
≥2.0	24 (57.1)	50 (61.0)		66 (58.9)	8 (66.7)		21 (56.8)	53 (60.9)		20 (55.6)	54 (61.4)	
Histological grade												
II	18 (42.9)	36 (43.9)	1.000	48 (42,9)	6 (50.0)	0.762	13 (35.1)	41 (47.1)	0.241	13 (36.1)	41 (46.4)	0.323
III	24 (57.1)	46 (56.1)		64 (57.1)	6 (50.0)		24 (64.9)	46 (52.9)		23 (63.9)	47 (53.4)	
Intrinsic subtype												
HER2E	15 (35.7)	39 (47.6)	0.252	47 (42.0)	7 (58.3)	0.362	15 (40.5)	39 (44.8)	0.696	19 (52.8)	35 (39.8)	0.232
lum B	27 (64.3)	43 (52.4)		65 (58.0)	5 (41.7)		22 (59.5)	48 (55.2)		17 (47.2)	53 (60.2)	
Lymph node status												
negative	15 (35.7)	34 (41.5)	0.566	43 (38.4)	6 (50.0)	0.538	12 (32.4)	37 (42.5)	0.322	15 (41.7)	34 (38.6)	0.840
positive	27 (64.3)	48 (58.5)		69 (61.6)	6 (50.0)		25 (67.6)	50 (57.5)		21 (58.3)	54 (61.4)	
Disease progression												
negative	22 (52.4)	68 (82.9)	0.001^‡^	80 (71.4)	10 (83.3)	0.509	26 (70.3)	64 (73.6)	0.826	29 (80.6)	61 (69.3)	0.269
positive	20 (47.6)	14 (17.1)		32 (28.6)	2 (16.7)		11 (27.9)	23 (26.4)		7 (19.4)	27 (30.7)	
pHER2^Y1248^												
negative				41 (36.6)	1 (8.3)	0.058	16 (43.2)	26 (29.9)	0.213	9 (25.0)	33 (37.5)	0.214
positive				71 (63.4)	11 (91.7)		21 (56.8)	61 (70.1)		27 (75.0)	55 (62.5)	
ErbB1/HER1												
negative	41 (97.6)	71 (86.6)	0.058				33 (89.3)	79 (90.8)	0.750	32 (88.9)	80 (90.9)	0.744
positive	1 (2.4)	11 (13.4)					4 (10.8)	8 (9.2)		4 (11.1)	8 (9.1)	
ErbB3/HER3												
negative	16 (38.1)	21 (25.6)	0.213	33 (29.5)	4 (33.3)	0.750				11 (30.6)	26 (29.5)	1.000
positive	26 (61.9)	61 (74.4)		79 (70.5	8 (66.7)					25 (69.4)	62 (70.5)	
ErbB4/HER4												
negative	9 (21.4)	27 (32.9)	0.214	32 (28.6)	4 (33.7)	0.744	11 (29.7)	25 (28.7)	1.000			
positive	33 (78.6)	53 (67.1)		80 (71.4)	8 (66.3)		26 (70.3)	62 (71.3)				
ER												
negative	15 (35.7)	42 (51.2)	0.128	50 (44.6)	7 (58.3)	0.544	15 (40.5)	42 (48.3)	0.440	21 (58.3)	36 (40.9)	0.112
positive	27 (64.3)	40 (48.8)		62 (55.4)	5 (41.7)		22 (59.5)	45 (51.7)		15 (41.7)	52 (59.1)	
PgR												
negative	24 (57.1)	50 (61.0)	0.703	65 (58.0)	9 (75.0)	0.358	24 (64.9)	50 (57.5)	0.549	24 (66.7)	50 (56.-8)	0.323
positive	18 (42.9)	32 (39.0)		47 (42.0)	3 (25.0)		13 (35.1)	37 (42.5)		12 (33.3)	38 (43.2)	

*Abbreviations: HER – human epidermal growth factor receptor; lum B – luminal B: estrogen receptor (ER) and/or progesterone receptor (PgR)-positive and HER2 positive; HER2E – ER- and PgR-negative and HER2-positive intrinsic subtype; pHER2^Y1248^ – tyrosine 1248-phosphorylated HER2.

†Bonferroni non-adjusted *P* values.

‡Significant *P* values (<Pc). Bonferroni correction for multiple comparisons Pc = 0.004 (0.05/12 - number of comparisons). Pearson chi square or Fisher exact test were used as appropriate.

**Figure 1:**
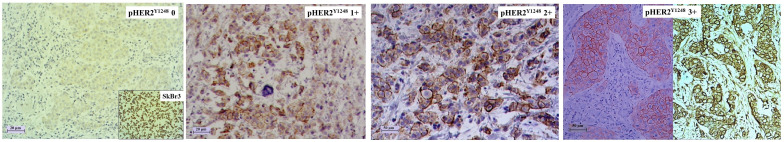
Immunohistochemical staining of tyrosine 1248-phosphorylated human epidermal growth factor receptor 2 (pHER2^Y1248^). From left to right: pHER2Y^1248^ 0, none or weak membranous staining; square – positive control of SkBr3 breast cancer (BC) cell line cells (overexpresses Her2 [Neu/ErbB-2] gene product stained for pHER2^Y1248^; pHER2^Y1248^ 1+, weak, fragmented membranous staining in >10% of tumor cells; pHER2^Y1248^ 2+, weak to moderate complete membrane staining in >10% of tumor cell, and pHER2^Y1248^ 3+, different variants of strong membranous staining in >10% of tumor cells.

**Figure 2:**
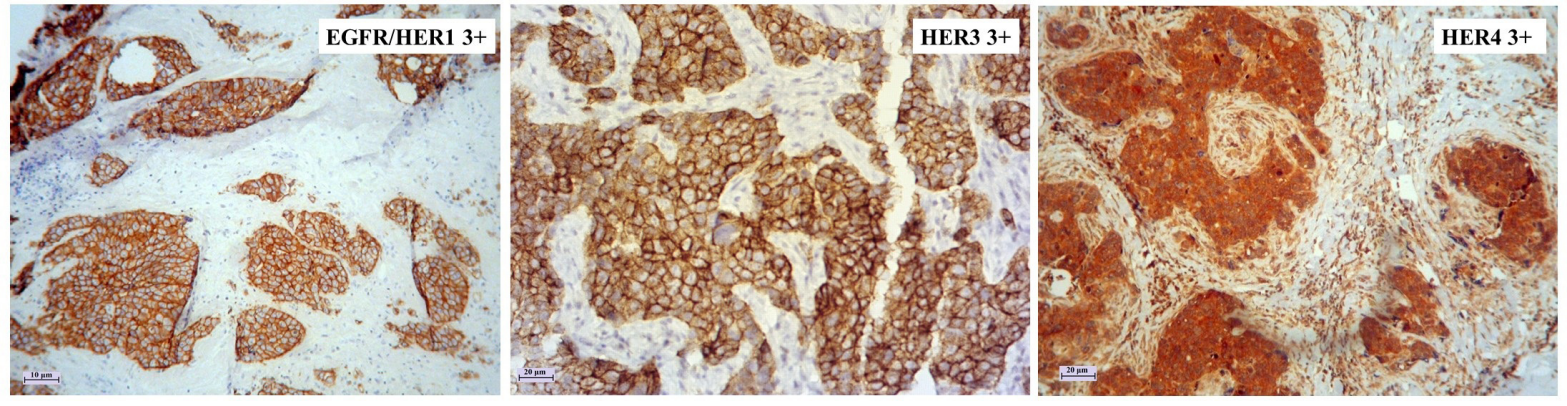
Immunohistochemical staining for human epidermal growth factor receptors (HER) analyzed in this study. Only strong immunohistochemical staining pattern is presented. From left to right: membranous EGFR/HER1 staining; membranous HER3 staining, and membranous/cytoplasmic HER4 staining.

The rates for HER1- and HER3-positive staining were 9.7 and 70.2, respectively (Table 2). Among HER1-positive patients, 4 showed moderate and 8 strong membranous immunoreactivity. Among HER3-positive patients, 54 (43.6%) showed moderate and 33 (26.6%) showed strong membranous staining. Predominantly cytoplasmic HER4-positive staining was identified in 88 (71.0%) patients (29.8% moderate and 41.2% strong positive staining), while 29.0% were negative (Figures 1 and 2).

### Association of expression of pHER2 and ErbB family members with clinicopathological parameters

The pHER2^Y1248^ status did not significantly correlate with tumor size (r = 0.037; *P* = 0.683), lymph node status (r = -0.056, *P* = 0.539), histological grade (r = -0.010, *P* = 0.912), intrinsic subtype (r = -0.113, *P* = 0.211), or the expression of ER (r = -0.147, *P* = 0.103) and PgR (r = -0.037, *P* = 0.683) (Table 2 and Table 3).

**Table 3:** Association of pHER2 and hormone receptors (ER and PgR) family members with clinicopathological prognostic features of breast cancer (BC) patients. The values are presented as frequencies and percentages*

	ER			PgR		
Variable	negative N = 57 (46.0)	positive N = 67 (54.0)	P^†^	negative N = 74 (59.7)	positive N = 50 (40.3)	P^†^
**Age of patients (years)**						
**<60**	37 (44.9)	40 (59.7)	0.582	43 (58.1)	34 (68.0)	0.346
**≥60**	20 (35.1)	27 (40.3)		31 (41.9)	16 (32.0)	
**Size of tumor (cm)**						
**<2.0**	18 (31.6)	32 (47.8)	0.098	25 (33.8)	25 (50.0)	0.093
**≥2.0**	39 (68.4)	35 (52.2)		49 (66.2)	25 (50.0)	
**Histological grade**						
**II**	21 (36.8)	33 (49.3)	0.204	29 (39.2)	25 (50.0)	0.270
**III**	36 (63.2)	34 (50.7)		45 (60.8)	25 (50.0)	
**Intrinsic subtype**						
**HER2E**	54 (94.7)	0 (0,0)	-	54 (73.0)	0 (0.0)	-
**luminal B**	3 (5.3)	67 (100.0)		20 (27.0)	50 (100.0)	
**Lymph node status**						
**negative**	24 (42.1)	25 (37.3)	0.713	29 (39.2)	20 (40.0)	1.000
**positive**	33 (57.9)	42 (62.7)		45 (60.8)	30 (60.0)	
**Disease progression**						
**negative**	38 (66.7)	52 (77.6)	0.226	51 (68.9)	39 (78.0)	0.309
**positive**	19 (33.3)	15 (22.4)		23 (31.1)	11(22.0)	
**pHER2^Y1248^**						
**negative**	15 (26.3)	27 (40.3)	0.128	24 (32.4)	18 (36.0)	0.703
**positive**	42 (73.7)	40 (59.7)		50(67.6)	32 (64.0)	
**ErbB1/HER1**						
**negative**	50 (87.7)	62 (92.5)	0.544	65 (87.9)	47 (94.0)	0.358
**positive**	7 (12.3)	5 (7.5)		9 (12.2)	3 (6.0)	
**ErbB3/HER3**						
**negative**	15 (26.3)	22 (32.8)	0.440	24 (32.4)	13 (26.0)	0.549
**positive**	42 (73.7)	45 (67.2)		50 (67.6)	37 (74.0)	
**ErbB4/HER4**						
**negative**	21 (36.8)	15 (22.4)	0.112	24 (32.4)	12 (24.0)	0.323
**positive**	36 (63.2)	52 (77.6)		50 (67.6)	38 (76.0)	
**ER**						
**negative**				54 (73.0)	3 (6.0)	<0.001^#^
**positive**				20 (270)	47 (94.0)	
**PgR**						
**negative**	54 (94.7)	20 (29.9)	<0.001^‡^			
**positive**	3 (5.3)	47 (70.1)				

*Abbreviations: HER1 – human epidermal growth factor receptor 1; HER3 – human epidermal growth factor receptor 3; HER4 – human epidermal growth factor receptor 4; luminal B – estrogen receptor (ER) and/or progesterone receptor (PgR)-positive and HER2 positive; HER2E – ER- and PgR-negative and HER2-positive intrinsic subtype; pHER2^Y1248^ – tyrosine 1248-phosphorylated human epidermal growth factor receptor 2.

†Bonferroni non-adjusted *P* values.

‡Significant *P* values (<Pc). Bonferroni correction for multiple comparisons Pc = 0.004 (0.05/12 - number of comparisons). Pearson chi square or Fisher exact test were used as appropriate.

pHER2^Y1248^-positive patients were more frequently HER3-positive, HER4-positive, hormone receptor-negative, and HER1-negative (Table 2). Thus, pHER2^Y1248^-positive patients were more commonly classified as luminal B (52.4%) than as HER2E (47.6%) (Table 2). pHER2^Y1248^ status significantly negatively and weakly correlated with younger age (r = -0.25, *P* = 0.005) and DP (r = -0.32; *P* < 0.001). However, after Bonferroni correction only the relationship with DP remained significant (Table 2). There were 70.7% of women younger than 60 years among pHER2^y1248^-positive patients and 45.2% among pHER2^y1248^-negative patients. DP was observed in only 17.1% of pHER2^Y1248^-positive and in 47.6% of pHER2^Y1248^-negative BCs (Table 2).

Among the standard clinicopathological characteristics, DP positively and weakly correlated with positive lymph node status (r = 0.31; *P* < 0.001) and higher histological grade (r = 0.25; *P* = 0.005) (Table 4). However, after the Bonferroni correction the relationship remained significant only for positive lymph node status (Table 4).

**Table 4:** The effect of clinicopathological variables on disease-free survival of HER2-positive breast cancer (BC) patients. The values are presented as frequencies and percentages*

	Disease progression		P^†^
	no	yes	
**Age stratification (years)**			0.535
**<60**	54 (60.0)	23 (67.6)	
**>60**	36 (40.0)	11 (32.4)	
**Tumor size (cm)**			0.066
**<2.0**	41 (45.6)	9 (26.5)	
**>2.0**	49 (54.4)	25 (73.5)	
**Histological grade**			0.008
**II**	46 (51,1)	8 (23.5)	
**III**	44 (48,9)	26 (76.5)	
**ER**			0.226
**negative**	38 (42.2)	19 (55.9)	
**positive**	52 (57.8)	15 (44.1)	
**PgR**			0.309
**negative**	51 (56.7)	23 (67.6)	
**positive**	39 (43.3)	11 (32.4)	
**Intrinsic subtype**			0.226
**luminal B**	54 (60.0)	16 (47.1)	
**HER2E**	36 (40.0)	18 (52.9)	
**Lymph node status**			0.001^‡^
**negative**	44 (48.9)	5 (14.7)	
**positive**	46 (51.1)	29 (85.3)	
**pHER2^Y1248^**			0.001^‡^
**negative**	22 (24.4)	20 (58.8)	
**positive**	68 (75.6)	14 (41.2)	
**HER1**			0.509
**negative**	80 (88.9)	32 (94.1)	
**positive**	10 (11.1)	2 (5.9)	
**HER3**			0.826
**negative**	26 (28.9)	11 (32.4)	
**positive**	64 (71.1)	23 (67.6)	
**HER4**			0.269
**negative**	29 (32.2)	7 (20.6)	
**positive**	61 (67.8)	27 (79.4)	

*Abbreviations: HER1 – human epidermal growth factor receptor 1; HER3 – human epidermal growth factor receptor 3; HER4 – human epidermal growth factor receptor 4; luminal B – estrogen receptor (ER) and/or progesterone receptor (PgR)-positive and HER2 positive; HER2E – ER, and PgR-negative and HER2-positive intrinsic subtype; pHER2^Y1248^ – tyrosine 1248- phosphorylated human epidermal growth factor receptor 2.

†Bonferroni non-adjusted *P* values.

‡Significant *P* values (<Pc). Bonferroni correction for multiple comparisons Pc = 0.005 (0.05/11 - number of comparisons). Pearson chi square or Fisher exact test were used as appropriate.

### Survival analyses

**Figure 3:**
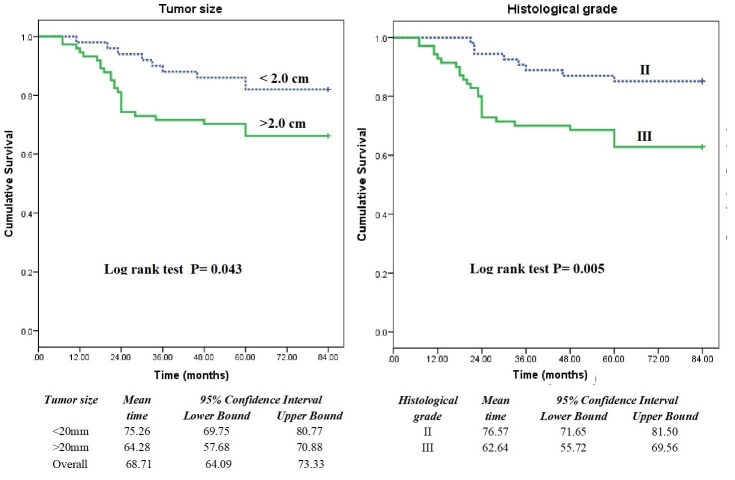
Kaplan-Meier estimates of five-year disease-free survival rate among breast cancer patients stratified according to tumor size (<2.0 cm vs >2.0 cm) and histological grade (II vs III).

**Figure 4:**
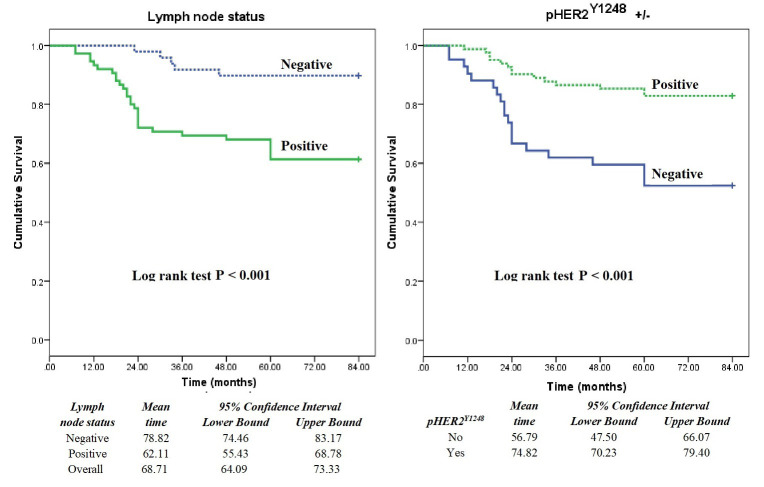
Kaplan-Meier estimates of five-year disease-free survival rate among breast cancer patients stratified according to lymph node status (LN- vs LN+) and pHER2^Y1248^-positive and pHER2^Y1248^-negative immunostaining pattern. Abbreviations: pHER2^Y1248^ – tyrosine 1248-phosphorylated human epidermal growth factor receptor 2.

Kaplan-Meier analysis showed that patients with larger tumor size (*P* = 0.043), higher histological grade (*P* = 0.005) (Figure 3), and positive lymph nodes (*P* < 0.001) were more likely to relapse after trastuzumab-based therapy (Figure 4). Nevertheless, after the Bonferroni correction only the effect of positive lymph node status remained significant (*P* < P_c_ [0.05/12 = 0.004]). Patients with DP were in most cases younger than 60 years (67.6%), had negative hormone receptor status (ER – 55.9%; PgR – 67.6%), and HER2E intrinsic subtype (52.9%). Notably, 58.8% of patients with DP showed pHER2^Y1248^-negative staining. In addition, the majority had HER1-negative (94.1%) and HER3-positive or HER4-postive status (67.6% and 79.4%, respectively, Table 4).

pHER2^Y1248^-negative status was significantly negatively associated with five-year DFS after trastuzumab treatment (*P* < 0.001, Figure 4). The mean DFS of pHER2^Y1248^-negative patients was 56.8 years, compared with 74.9 years in pHER2^Y1248^-positive patients (Figure 4). Moreover, univariate Cox regression analysis revealed that the pHER2^Y1248^-negative status was the most significant prognostic factor for a worse five-year DFS (HR 3.4, 95% CI 1.7-6.8, *P* < 0.001<P_c_ [0.05/11 = 0.005]) (Table 5). Independent prognostic power of pHER2^Y1248^-negative status in predicting DFS was further confirmed with multivariate Cox regression analysis (HR 3.6, 95% CI 1.8-7.2, *P* < 0.001).

**Table 5:** Predictors of five-year disease-free survival on univariate and multivariate analysis of breast cancer (BC) HER2-positive patients*

	Univariate		Multivariate	
Variable	HR (95% CI)	Cox P^†^	HR (95% CI)	Cox P^§^
**pHER2^Y1248^ (negative vs positive)**	3.4 (1.7-6.8)	<0.001^‡^	3.6 (1.8-7.2)	<0.001
**Lymph node (positive vs negative)**	4.6 (1.8-11.9)	0.002^‡^	3.6 (1.3-9.8)	0.014
**Histological grade (III vs II)**	2.96 (1.3-6.5)	0.007	2.0 (0.9-4.6)	0.116
**Tumor size (≥2.0 cm vs <2.0 cm)**	2.1 (1.0-4.6)	0.050		
**Intrinsic subtypes** **(Luminal B vs HER2E)**	0.6 (0.3-1.2)	0.168		
**Age (<60 vs ≥60 years)**	1.3 (0.6-2.6)	0.539		
**ER (negative vs positive)**	1.6 (0.8-3.2)	0.159		
**PgR (negative vs positive)**	1.6 (0.0-3.2)	0.213		
**HER1 (negative vs positive)**	1.8 (0.4-7.7)	0.405		
**HER3 (positive vs negative)**	0.9 (0.4-1.83)	0.755		
**HER4 (positive vs negative)**	1.7 (0.7-3.9)	0.220		

*Abbreviations: CI – confidence interval; HR – hazard ratio; pHER2^Y1248^ – tyrosine 1248–phosphorylated human epidermal growth factor receptor 2; HER1 – human epidermal growth factor receptor 1; HER3 – human epidermal growth factor receptor 3; HER4 – human epidermal growth factor receptor 4; Luminal B – estrogen receptor (ER) and/or progesterone receptor (PgR)-positive and HER2 positive; HER2E – ER, and PgR-negative, and HER2-positive intrinsic subtype; Cox P – *P* values for Cox regression analysis;

†Bonferroni non-adjusted *P* values.

‡Significant *P* values (<P_c_ = 0.005 in univariate Cox regression analysis). Bonferroni correction for multiple tests in univariate Cox regression analysis P_c_ = 0.005 (0.05/11– number of comparisons).

**§**Bonferroni correction not applicable.

**Figure 5:**
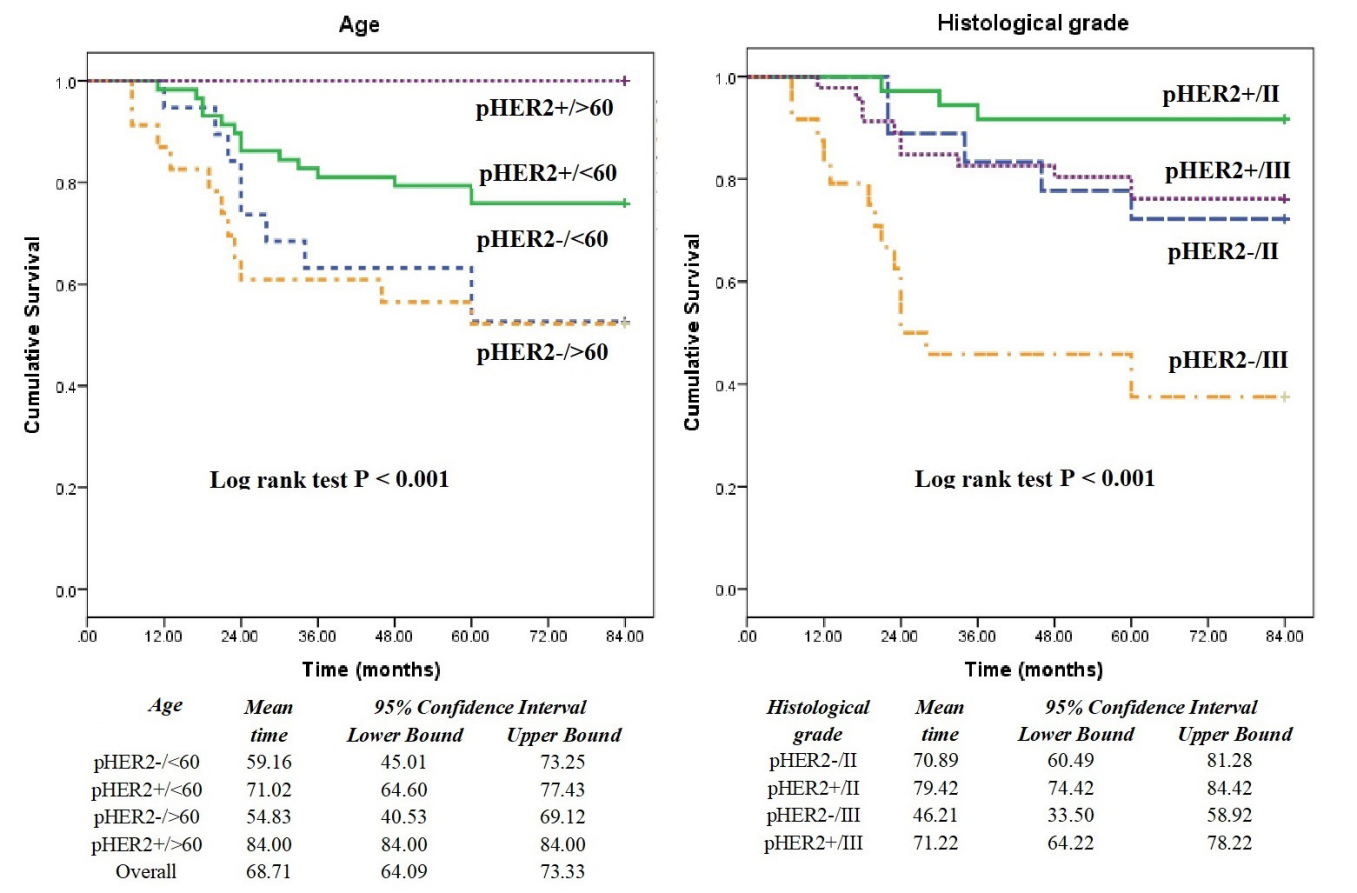
Kaplan-Meier estimates of five-year disease-free survival rate of breast cancer patients with positive and negative tyrosine 1248-phosphorylated human epidermal growth factor receptor 2 (pHER2^Y1248^) immunohistochemical status stratified according to age subgroups at surgery (<60 years vs >60 years) and tumor histological grade (II vs III).

**Figure 6:**
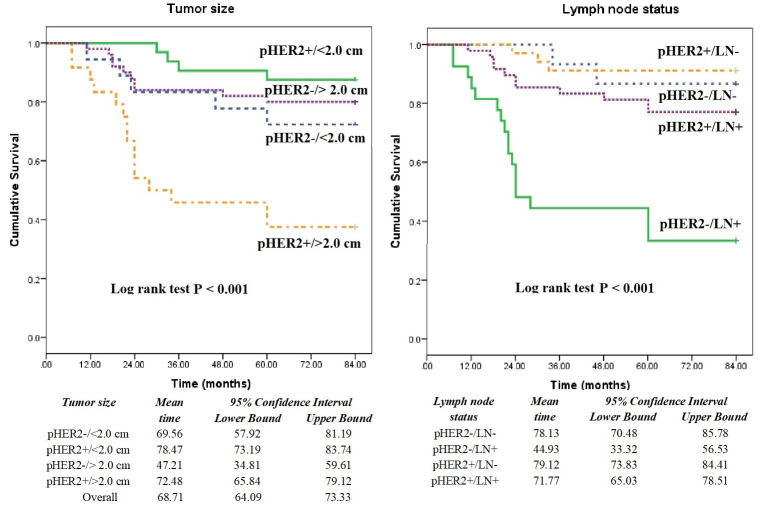
Kaplan-Meier estimates of five-year disease-free survival rate of breast cancer patients with positive and negative tyrosine 1248-phosphorylated human epidermal growth factor receptor 2 (pHER2^Y1248^) immunohistochemical status stratified according to tumor size (<2.0 cm vs >2.0 cm) and lymph node status (LN+ vs LN-).

The average DFS of pHER2^Y1248^-negative patients older than 60 years, with higher histological tumor grade, a larger tumor, or positive lymph nodes was 54.8, 47.2, 46.2, and 44.9 months, respectively (Figure 5 and 6).

In univariate Cox regression analysis, positive lymph nodes (HR 4.6, 95% CI 1.8-11.9, *P* = 0.002<P_c_ [0.05/11 = 0.005]) and higher histological grade (HR 2.96, 95% CI 1.3-6.5, *P* = 0.007) were both negatively associated with five-year DFS (Table 5). However, multivariate Cox regression analysis confirmed only positive lymph node status (HR 3.6, 95% CI 1.3-9.8, *P* = 0.014) as an additional indicator of a worse five-year DFS (Table 5).

**Figure 7:**
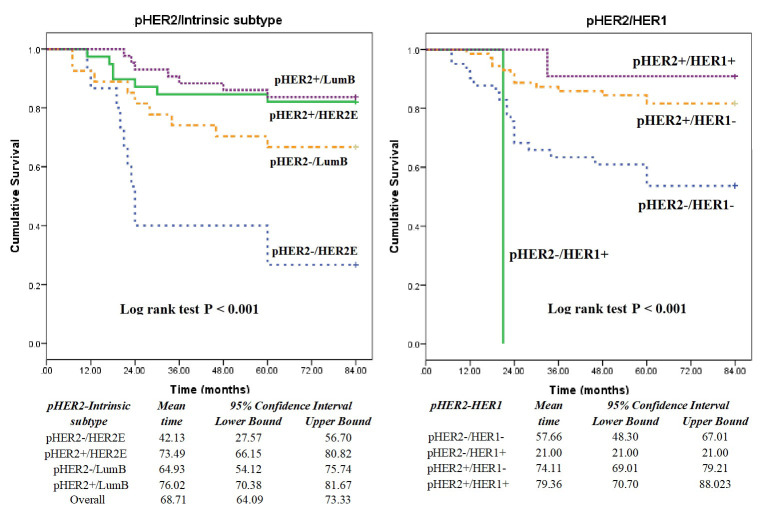
Kaplan-Meier estimates of five-year disease-free survival rate of breast cancer patients with positive and negative tyrosine 1248-phosphorylated human epidermal growth factor receptor 2 (pHER2^Y1248^) immunohistochemical status stratified according to tumor intrinsic subtype (LumB vs HER2E), and HER1 immunohistochemical staining (HER1- vs HER1+). Abbreviations: LumB – estrogen receptor (ER)- and/or progesterone receptor (PgR)-positive and HER2-positive; HER2E – ER- and PgR-negative, and HER2-positive intrinsic subtype.

**Figure 8:**
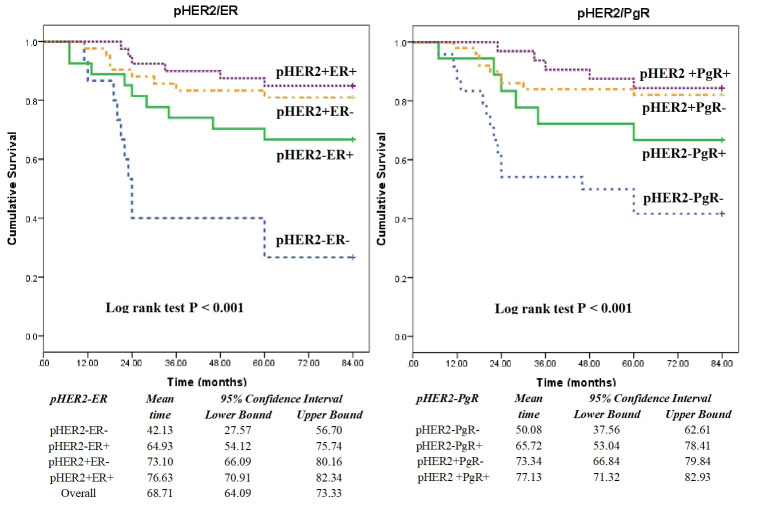
Kaplan-Meier estimates of five-year disease-free survival rate of breast cancer patients with positive and negative tyrosine 1248-phosphorylated human epidermal growth factor receptor 2 (pHER2^Y1248^) immunohistochemical status stratified according to ER (ER- vs ER+) and PgR (PgR- vs PgR+) immunohistochemical staining. Abbreviations: ER – estrogen receptor, PgR – progesterone receptor.

**Figure 9:**
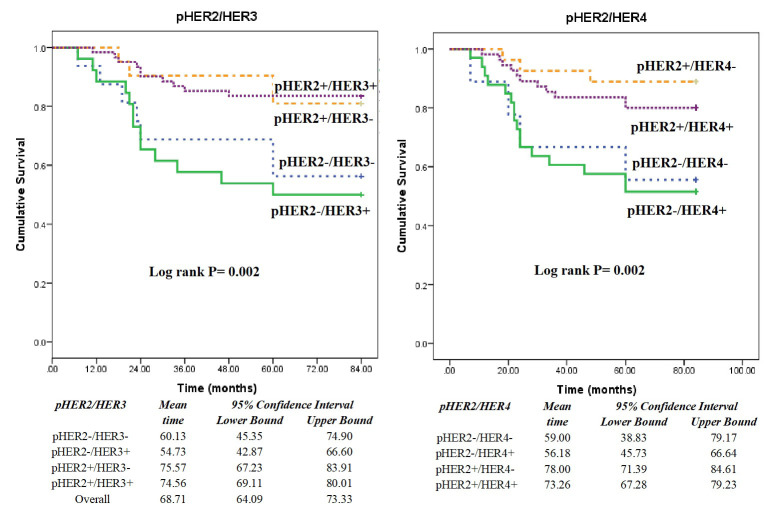
Kaplan-Meier estimates of five-year disease-free survival rate of breast cancer (BC) patients with positive and negative tyrosine 1248-phosphorylated human epidermal growth factor receptor 2 (pHER2^Y1248^) immunohistochemical status stratified according to HER3 (HER3- vs HER3+) and HER4 (HR4- vs HR4+) immunohistochemical staining. Abbreviations: HER – epidermal growth factor receptor.

Univariate Cox regression analysis revealed that the intrinsic subtype, hormone receptor status, and HER3 and HER4 staining were not significantly related to DFS (Table 5). Nevertheless, when these biomarkers were stratified according to pHER2^Y1248^ status, Kaplan-Meier analysis showed significant differences in five-year DFS (*P* < P_c_ [0.05/11 = 0.005]) (Figure 6). Thus, pHER2^Y1248^-negative patients with HER2E intrinsic subtype, as well as those with HER1-negative staining (Figure 7) or negative hormone receptors (Figure 8) had a worse five-year DFS. The same was true for pHER2^Y1248^-negative patients with the coexpression of HER3+ or HER4+ (Figure 9).

## DISCUSSION

In our study, pHER2^Y1248^ predicted sensitivity to trastuzumab and a better five-year DFS regardless of any other prognostic parameter. Additionally, pHER2^Y1248^-negative and lymph node-positive status were the only negative prognostic factors for five-year DFS of HER2-positive BC patients. Furthermore, non-phosphorylated HER2^Y1248^ was a strong predictor of resistance and poor five-year DFS in combination with any clinicopathological parameter: in patients older than 60 years or those with positive lymph nodes, larger tumor size, or higher histological stage. This was especially true in patients with HER2E-type tumor or those negative for EGFR/HER1 and/or positive for HER3 or HER4.

The expression of pHER2^Y1248^ receptors was identified in 66.1% of our patients, consistent with 68% of HER2-positive BC cases in the report by Cicenas et al (13). A relatively high expression rate of pHER2Y^1248^ in HER2-positive BC was also found by other researchers (14-19). However, smaller rates (12%-38.2%) were also reported (20-27). The discrepancies in pHER2^Y1248^ expression might be explained by differences in assays sensitivity, scoring systems, cut-off values used for the evaluation of pHER2 expression, and likely difference in the degree of phosphorylation/activation of HER2 in biologically distinct BC patient cohorts. Thus, for example, HER2 overexpression with concomitant pHER2^Y1248^-positive status was considerably less common in ER+ BCs than previously believed (28).

Taniyama et al (19) showed pHER2^Y1248^ expression to be highly specific for HER2 gene amplification, advanced tumor stage, and poor DFS of patients with invasive ductal BC. Furthermore, the total level of pHER2^Y1248^ is considered to be physiologically more important than the overall number of HER2 present in the cancer tissue (20). Importantly, not all BC patients with pHER2^Y1248^ also overexpressed HER2, which indicates signaling activation distinct from the classical pathway involving homo-dimerization and hetero-dimerization with other ErbB family members (13,29). Previous studies also indicate that phosphorylation of HER2 at Y1248 occurs only when HER2 is dimerized and activated irrespective of its overexpression status (11,24).

Phosphorylation of HER2 leading to its activation is closely associated with subsequent signaling transduction to its downstream targets that mediate cellular proliferation, migration, or adhesion, thus profoundly affecting DP and overall prognosis (30-38).

In our study, patients with positive pHER2^Y1248^ had a nearly three times lower risk of DP after trastuzumab-based treatment and a better five-year DFS compared with patients with negative pHER2^Y1248^, of whom 58% relapsed.

Similarly, Hudelist et al (39) reported that pHER2^Y1248^-positive staining was the only covariate predicting the benefit of trastuzumab-based treatment in metastatic BC patients exhibiting moderate or strong HER2 overexpression (39). Notably, in their cohort, the progression-free survival to trastuzumab-based treatment was more than doubled in pHER2^Y1248^-positive compared with pHER2^Y1248^-negative BC (39).

Better response of pHER2^Y1248^-positive BC to trastuzumab-based therapy was also reported by Giuliani et al (27). Besides, Dokmanovic et al (40) reported that pHER2^Y1248^-positive staining in HER2-positive BC correlated with increased trastuzumab response in the neoadjuvant settings. Notably, the majority of the patients with DP or residual disease after neoadjuvant trastuzumab treatment were pHER2^Y1248^-negative, whereas 4/5 patients with complete or near-complete pathological remission were pHER2^Y1248^-positive (40).

A trend toward increased sensitivity to trastuzumab treatment was also confirmed in experiments on HER2-overexpressing BC cell lines (9). In the study by Ginestier et al (9), the majority of cell lines (8/10) sensitive to trastuzumab treatment were pHER2^Y1248^-positive, while 4/6 resistant cell lines were either weakly positive or negative. Furthermore, Diemeier et al (41) reported that the dominant growth-inhibitory effect of trastuzumab on trastuzumab-sensitive BT474 and SK-BR-3 BC cells lines was associated with its ability to induce HER2 phosphorylation at Y1248.

Contrary to this, Kurebayashi et al (18) reported a worse prognostic effect of pHER2^Y1248^ in HER2-positive BC patients treated with trastuzumab and chemotherapy. They hypothesized that the worse outcome of such patients might be related to the inability of trastuzumab to inhibit DP through ligand-dependent HER2-HER3 hetero-dimerization (42-44).

Interestingly, Cheng et al (46) found no association between pHER2^Y1248^ and the response to trastuzumab-containing neoadjuvant therapy in the pre-surgical setting. No association of pHER2^Y1248^ and trastuzumab treatment of HER2-positive BC patients was also reported by Dębska-Szmich et al (26). In other studies, pHER2^Y1248^-positive staining was associated with a worse prognosis in primary, treatment-naive BC patients (13,14,16,20,24,47,48).

In our study, the majority of HER2-positive patients with DP exhibited hormone receptor-negative, EGFR/HER1-negative immunostaining, and positive HER3 and/or HER4 status. In a study by DiGiovanna et al (14), pHER2^Y1248^ expression was significantly associated with a positive expression of EGFR/HER1. A worse outcome was observed in patients who were either positive for all three biomarkers examined (pHER2^Y1248^, EGFR/HER1, and HER2) or positive only for EGFR/HER1 and HER2 but negative for pHER2^Y1248^ (14). Significantly higher expression of pHER2^Y1248^ among patients with high EGFR/HER1 levels was also reported by Cicenas et al (13). However, despite the positive correlation between the pHER2^Y1248^-positive status and EGFR/HER1, its overall expression did not differ between pHER2^Y1248^-positive and pHER2^Y1248^-negative patients. In addition, pHER2^Y1248^ expression positively correlated with HER2 and inversely correlated with hormone receptors, HER3, and HER4. Hormone receptors and HER4 levels were significantly lower in patients with higher pHER2^Y1248^ (13). Contrary to these reports, Kurebayashi et al (18) reported no significant correlation of pHER2^Y1248^ with EGFR/HER1 and HER4 expression in HER2-positive BC patients. However, pHER2^Y1248^ significantly correlated with HER2 and HER3 expression (18).

In our study, a worse five-year DFS was detected in patients with pHER2^Y1248^-negative and either hormone receptor-negative or HER1-negative status and in patients with pHER2^Y1248^-negative immunostaining co-expressed with positive HER3 or HER4. Our results suggest that in such cases phosphorylation occurred at other phosphorylation sites and heterodimerization activated other signaling pathways.

There are several limitations to our study. The sample size was moderate, encompassing BC patients with specific biomarker signatures. Second, the majority of BC in our patient cohort exhibited a HercepTest score of 3+, while the number of HER2 equivocal (2+) patients was limited. Third, all patients received adjuvant trastuzumab therapy so future studies should assess the prognostic value of pHER2^Y1248^ in the neoadjuvant settings.

In conclusion, our results indicate that the pHER2^Y1248^-negative status of HER2-overexpressing BC represents a strong independent predictor of tumor resistance to trastuzumab-based therapy and poor five-year DFS rate irrespective of other biomarkers and clinicopathological variables tested. Further studies are needed to investigate the predictive value of the pHER2^Y1248^ in a larger cohort of HER2-positive BC patients.
